# 

**Published:** 1918-01-19

**Authors:** 


### CONTENTS.

| | PAGE |
|---|---|
| **University Developments** ... ... ... ... ... | 325 |
| **The "Rewa" Outrage** ... ... ... ... | 326 |
| **Hospital and Institutional News** ... ... ... | 327 |
| The New Director-General Army Medical Services—Sir Robert Arundell Hudson, G.B.E.—A Notable Masonic Event—Death of the Late Chairman of Mount Vernon Hospital—The Case Against the Pensions Minister—Capitation Grants for Military Out-patients—The Imperial War Exhibition—Extension by Hut—Gaza Hospital—The Sale of Secret Remedies in France—Salvationists in France—A Literary Point—Institution Fire Precautions—Generous Assistance to Maternity and Child-welfare Schemes—Abertillery Hospital—The Importance of the Crèche—This Week's Drug Market. | |
| **Deductions from the World War** ... ... | 331 |
| The Two Pathways: Which will the World Choose? | |
| **Sir Alfred Keogh, G.C.B. M.D.** ... ... ... | 333 |
| Rector of the Imperial College of Science and Technology. | |
| **Glimpses Backward** ... ... ... ... ... ... | 335 |
| II. Some Masters of Mediæval Surgery. | |
| **Nursing Affairs in Ireland** ... ... ... ... | 336 |
| More Enemy War-Cries: Lay Control. | |
| **The Editor's Letter-Box** ... ... ... ... | 336 |
| The Orphan Working School. | |
| **The Matrons' and Sisters' Department** ... | 337 |
| The Growth of the Nursing Profession: The Habit of Talking Too Much. | |
| Round the Hospitals. | |
| The Soldier and the Nurse—The Essential Spirit to Victory—Emptying Drawers for the Red Cross—More Strength for College Council—Larger Salaries at Three Great Hospitals—How Frozen Meat Should be Cooked. | |
| **Some Hospital Types of British Nursing** ... | 339 |
| The Home Sister of my Alma Mater. | |
| **The Heating of Hospitals** ... ... ... ... ... | 341 |
| XIII. Middlesex Hospital. | |
| **Mr. H. J. Paterson, F.R.C.S., with his Camera** | 343 |
| Some Remarkable Views. | |
| **Personalia** ... ... ... ... ... ... ... | 343 |
| **Matrons' and Sisters' Appointments** ... ... | 344 |
| **Gifts and Bequests** ... ... ... ... ... ... | 344 |
| **Institutional Needs** ... ... ... ... ... ... | 344 |
| **The Institutional Worker** ... ... (Suppl.) | 1 |
| The Ontario Military Hospital, Orpington. | |

